# Novel molecular changes induced by *Nrg1* hypomorphism and *Nrg1*-cannabinoid interaction in adolescence: a hippocampal proteomic study in mice

**DOI:** 10.3389/fncel.2013.00015

**Published:** 2013-02-26

**Authors:** Jarrah R. Spencer, Keturah M. E. Darbyshire, Aurelie A. Boucher, Mohammed A. Kashem, Leonora E. Long, Iain S. McGregor, Tim Karl, Jonathon C. Arnold

**Affiliations:** ^1^Discipline of Pharmacology, University of SydneySydney, NSW, Australia; ^2^Brain and Mind Research Institute, University of SydneySydney, NSW, Australia; ^3^School of Psychology, University of SydneySydney, NSW, Australia; ^4^Neuroscience Research AustraliaRandwick, NSW, Australia; ^5^Schizophrenia Research InstituteDarlinghurst, NSW, Australia; ^6^School of Medical Sciences, University of New South WalesKensington, NSW, Australia

**Keywords:** *Nrg1*, THC, mouse, hippocampus, schizophrenia, proteomics

## Abstract

Neuregulin 1 (*NRG1*) is linked to an increased risk of developing schizophrenia and cannabis dependence. Mice that are hypomorphic for *Nrg1* (*Nrg1* HET mice) display schizophrenia-relevant behavioral phenotypes and aberrant expression of serotonin and glutamate receptors. *Nrg1* HET mice also display idiosyncratic responses to the main psychoactive constituent of cannabis, Δ^9^-tetrahydrocannabinol (THC). To gain traction on the molecular pathways disrupted by *Nrg1* hypomorphism and *Nrg1*-cannabinoid interactions we conducted a proteomic study. Adolescent wildtype (WT) and *Nrg1* HET mice were exposed to repeated injections of vehicle or THC and their hippocampi were submitted to 2D gel proteomics. Comparison of WT and *Nrg1* HET mice identified proteins linked to molecular changes in schizophrenia that have not been previously associated with *Nrg1*. These proteins are involved in vesicular release of neurotransmitters such as SNARE proteins; enzymes impacting serotonergic neurotransmission, and proteins affecting growth factor expression. *Nrg1* HET mice treated with THC expressed a distinct protein expression signature compared to WT mice. Replicating prior findings, THC caused proteomic changes in WT mice suggestive of greater oxidative stress and neurodegeneration. We have previously observed that THC selectively increased hippocampal NMDA receptor binding of adolescent *Nrg1* HET mice. Here we observed outcomes consistent with heightened NMDA-mediated glutamatergic neurotransmission. This included differential expression of proteins involved in NMDA receptor trafficking to the synaptic membrane; lipid raft stabilization of synaptic NMDA receptors; and homeostatic responses to dampen excitotoxicity. These findings uncover novel proteins altered in response to *Nrg1* hypomorphism and *Nrg1*-cannabinoid interactions that improves our molecular understanding of *Nrg1* signaling and *Nrg1*-mediated genetic vulnerability to the neurobehavioral effects of cannabinoids.

## Introduction

Neuregulin 1 (*Nrg1*) is a neurotrophic factor that mediates its effects by binding ErbB receptor tyrosine kinases. *Nrg1* regulates axonal guidance, myelination, and GABAergic and glutamatergic neurotransmission (Mei and Xiong, 2008). The human *NRG1* gene has been linked to schizophrenia by genetic studies (Stefansson et al., 2002; Ayalew et al., 2012) and altered expression of *NRG1* isoforms has been measured in schizophrenia patients (Hashimoto et al., 2004; Chong et al., 2008; Marballi et al., 2012; Weickert et al., 2012). *NRG1* variants have been associated with dysfunction in a number of schizophrenia-relevant “endophenotypes” including sensorimotor gating as measured by prepulse inhibition of startle (PPI) (Hong et al., 2008; Roussos et al., 2011; Greenwood et al., 2012) and working memory (Chong et al., 2008).

Use of transgenic murine models can be useful in exploring the role of *Nrg1* in molecular neurobiology and behavior. The most extensively studied mouse model of *Nrg1* dysfunction is the *Nrg1* transmembrane heterozygous (*Nrg1* HET) mouse which exhibits locomotor hyperactivity and protocol-dependent PPI deficits (Stefansson et al., 2002; Karl et al., 2007; Spencer et al., 2012). These mice display altered anxiety profiles, inhibited preference for social novelty and increased levels of aggressive social interaction as well as impaired performance in novel object recognition and fear conditioning paradigms (Karl et al., 2007; O'Tuathaigh et al., 2007, 2008; Duffy et al., 2010; Desbonnet et al., 2012). Hypo-phosphorylation of the NR2B subunit of the NMDA receptor is observed in *Nrg1* HET mice (Bjarnadottir et al., 2007). Together, these findings provide some clues of the molecular and neurobiological alterations that mediate the aberrant behavioral phenotypes exhibited by *Nrg1* HET mice.

Adolescence is particularly relevant to schizophrenia given the onset of the disorder typically occurs in late adolescence. During adolescence there exists significant synaptic pruning and a shift between utilization of mesolimbic and mesocortical areas of the brain which indicates a high level of neural development during this period (Giedd et al., 1999; Spear, 2000; Casey et al., 2008). *Nrg1* HET mice display developmentally-specific neurobiological and behavioral phenotypes, for example, adolescent *Nrg1* HET mice have reduced 5-HT_2A_ receptor expression in the insular and cingulate cortices (Long et al., 2013) in contrast to the global increase in 5HT_2A_ receptor expression observed in adult *Nrg1* HET mice relative to controls (Dean et al., 2008). *Nrg1* HET mice display an enhanced stress-induced release of corticosterone relative to wildtype (WT) controls at 3–4 months of age, an effect that disappears by 5–6 months (Chesworth et al., 2012). Together these findings point toward the *Nrg1* HET mouse being a particularly suitable model for demonstrating a role for *Nrg1* in developmental stage-specific neurobehavioral alterations.

Drug dependence and schizophrenia are comorbid disorders that may have common genetic and neurobiological substrates. Genetic vulnerability is thought to explain why only a subset of cannabis users become dependent on cannabis or develop psychosis. A recent study demonstrated that *NRG1* increased the risk of cannabis dependence in African-Americans (Han et al., 2012). We have shown *Nrg1* HET mice display distinct schizophrenia-relevant neurobehavioral responses to cannabinoids, including the main psychoactive constituent of cannabis, Δ^9^-tetrahydrocannabinol (THC). Acute cannabinoid exposure promoted PPI facilitation in *Nrg1* HET mice but PPI deficits in WT mice (Boucher et al., 2007a, 2011). *Nrg1* genotype also modulated tolerance to the effects of cannabinoids, with *Nrg1* HET mice developing tolerance more rapidly to locomotor suppression and hypothermia than WT mice, but conversely showing a lack of tolerance to cannabinoid-induced anxiety unlike WT mice (Boucher et al., 2011). The acute and repeated effects of cannabinoids correlate with selective changes in Fos transcription factor expression in the lateral septum of *Nrg1* HET mice that were not observed in WT mice (Boucher et al., 2007b, 2011). In adolescence *Nrg1* modulated the effects of repeated THC exposure on the expression of neurotransmitter receptors relevant to the pathophysiology of schizophrenia (i.e., CB_1_, NMDA, and 5-HT_2A_ receptors) (Long et al., 2013).

The hippocampus may be an important region for *Nrg1*-cannabinoid interactions as both endocannabinoid and *Nrg1*-ErbB systems are highly expressed in this brain region (Herkenham et al., 1990; Tsou et al., 1998; Vullhorst et al., 2009). We have observed increased brain transcriptional activity in the lateral septum at baseline and following cannabinoid exposure in *Nrg1* HET mice, both of which might reflect downstream effects of aberrant activity in the hippocampus as part of the septohippocampal system. Therefore, molecular changes in the hippocampus may subserve the distinct neurobehavioral phenotypes displayed by *Nrg1* HET mice as well as their altered response to THC. Of particular interest is our observation that adolescent THC–treated *Nrg1* HET mice display increased NMDA receptor expression in the hippocampus, something not observed in THC-treated WT mice (Long et al., 2013). Here we aim to gain some traction on the molecular mechanisms involved in the aberrant phenotypes exhibited by *Nrg1* HET mice at baseline and when exposed to THC using a proteomic approach which allows us to detect changes in hundreds of different proteins in the hippocampus.

## Materials and methods

### Animals and drug treatment

At the commencement of the study adolescent male *Nrg1* HET mice and WT littermates (C57/BL6 background strain) were at an age of post-natal day (PND) 31 ± 2. The study was restricted to male mice as male *Nrg1* HET mice appear more vulnerable to the effects of cannabinoids (Long et al., 2010). Mice were pair-housed at Neuroscience Research Australia with limited environmental enrichment [certified polycarbonate mouse igloo (Bioserv, USA) and a metal ring in the cage lid] under a 12 h light/dark schedule (lights on 08:30 h) and genotyped as previously detailed (Karl et al., 2007). Food and water were available *ad libitum*. THC (THC Pharm GmbH, Germany) was suspended in a 1:1:18 mixture of ethanol:Tween 80:0.9% saline and injected intraperitoneally at a volume of 10 ml/kg. Mice were injected daily with either 10 mg/kg of THC or vehicle for 21 days. During this time mice were repeatedly behaviorally tested, the results of which are published elsewhere (Long et al., 2013). Two days following the completion of treatment, the mice (*n* = 8) were euthanized by cervical dislocation, with both hippocampi dissected out and snap frozen on dry ice for proteomic analysis. Research and animal care procedures were approved by the University of New South Wales Animal Care and Ethics Committee and were in accordance with the Australian Code of Practice for the Care and Use of Animals for Scientific Purposes.

### Protein extraction

Protein extraction was performed using a protocol optimized for cytosolic proteins (Quinn et al., 2008). Hippocampal tissue was homogenized in buffer consisting of 7 M urea, 2 M thiourea, 1% C7bZO and 40 mM Tris, sonicated and pelleted. The supernatant was reduced and alkylated in 5 mM tributylphosphine (TBP) and 10 mM acrylamide monomer and quenched using 10 mM dithiothreitol (DTT). The mixture was acidified to pH 6.0 using concentrated citric acid and precipitated with acetone. The precipitate was pelleted, air-dried and resuspended in 7 M urea, 2 M thiourea and 1% C7bZO.

### 2D gel electrophoresis

Sample protein concentration was determined using the Bradford Protein Assay (Thermoscientific, USA). Immobilized pH gradient strips (IPG strips; 11 cm, pH 4–7) were rehydrated with samples containing 600 μg protein, and samples were separated by isoelectric point (pI). Strips were equilibrated in SDS equilibration buffer and loaded onto SDS-PAGE gradient gels (8–16%, 10 × 15 cm) and separated by molecular weight using an ElectrophoretIQ3 system (30 mA/gel, 25°C for 110 min; Proteome Systems, Australia). Gels were fixed with methanol [25% (v/v)] and acetic acid [10% (v/v)] and visualized using Flamingo Fluorescent gel stain (BioRad, USA).

### Image analysis

Gels were analyzed using Phoretix 2D Expression software (Non-linear Dynamics Ltd, UK). Averaged gels were created for each experimental group and averaging parameters were set at 70%. Single factor ANOVAs (*p* < 0.05) of spot volume were performed to determine the effect of genotype in vehicle-treated animals, the effect of THC administration in WT mice and the effect of THC administration in *Nrg1* HET mice.

### Mass spectrometry and protein identification

Protein spots that were identified as significantly altered were digested in 12.5 ng/mL trypsin (Roche, USA) and 25 nM NH_4_HCO_3_/0.1% trifluroacetic acid and purified using C_18_ purification tips (Eppendorf, Germany) before being eluted in 3 μL of matrix solution. Spots were analyzed using an Applied Biosystems QSTAR MALDI-TOF mass spectrometer (Australian Proteome Analysis Facility, University of Sydney). MALDI spectra were matched against the Swiss-Prot database using the MASCOT search engine with matches determined by molecular weight search score (MWS) and sequence coverage in conjunction with pI and molecular weight as estimated from gels.

### Immunoblotting

Ten ug of protein per lane was separated by electrophoresis using 10% precast NuPage gels (Invitrogen, USA) and run at 110 V for 2 h. The samples were transferred to PVDF membranes. Membranes were sequentially incubated with Syntaxin-1A antibody (Sigma Aldrich, USA 1:1500) then swine anti-rabbit secondary antibody (DAKO, Australia, 1:200) and rabbit Peroxidase-Anti-Peroxidase (Sigma-Aldrich, USA 1:200) and DAB (DAKO, Australia). Membranes were stripped using Re-blot plus strong antibody stripping solution (Millipore, Australia) and incubated sequentially with Abcam rat monoclonal YL1/2 α-tubulin (TUBA) antibody (Sapphire Biosciences Pty Ltd, Australia, 1:) then anti-rat IgG (HþL) horseradish peroxidase conjugate (Santa Cruz Biotechnology Inc, USA) and visualized using a Syngene G:Box.

## Results

**Figure 1:**
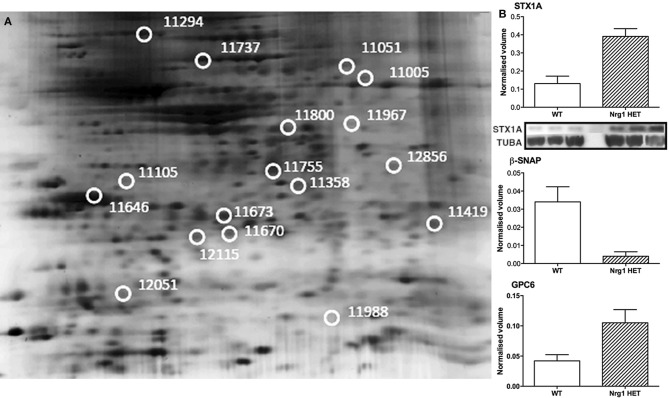
**Adolescent hippocampal proteomics comparing adolescent vehicle-treated WT and *Nrg1* HET mice. (A)** Representative 2DE gel images showing positions of significantly altered protein spots [the horizontal axis split by isoelectric point, vertical axis split by molecular weight; STX1A (spot 11755); TPH1 (spot 11967); β-SNAP (spot 11358); SPARC (spot 11646); GPC6 (spot 11294)]; and **(B)** Exemplar normalized volumes of altered proteins i.e., STX1A, β-SNAP and GPC6. The inset for STX1A shows immunoblots against STX1A and loading control alpha-tubulin (TUBA), confirming greater expression of STX1A in *Nrg1* HET versus WT mice.

Here we present the results of a hippocampal proteomic study conducted on adolescent *Nrg1* HET mice and WT control mice treated with or without THC. The averaged gels for WT vehicle, WT THC, *Nrg1* HET vehicle and *Nrg1* HET THC contained 870, 821, 761, and 742 spots respectively. 26 spots were significantly different between WT vehicle and *Nrg1* HET vehicle mice. Of these spots, 17 proteins were identified using MALDI-TOF MS and the fold changes from control WT mice are listed in Table 1. Figure 1A shows a representative 2D gel image of protein expression in the hippocampus of a WT mouse administered vehicle. Normalized spot volumes are depicted for three representative proteins are shown in Figure 1B, i.e., syntaxin 1A (STX1A), beta-soluble N-ethylmaleimide-sensitive factor attachment protein (β-SNAP) and glypican 6 (GPC6). Western blotting results confirmed *Nrg1* HET mice displayed increased expression of STX1A compared to WT mice (see Figure 1B).

**Table 1:** **Adolescent hippocampal proteomics comparing adolescent vehicle-treated WT and *Nrg1* HET mice**.

**Spot number**	**Protein name**	**Abbreviation**	**UniProt accession number**	**PI**	**Mass (Da)**	**MWS**	**No. of peptides matched**	**% seq cover**	**Fold change**	***T*-test (p)**
**VESICLE FUNCTION PROTEINS**										
11755	Syntaxin-1A	STX1A	O35526	5.14	33,054	63	5	20	2.996	0.00123
11358	Beta-soluble NSF attachment protein	β-SNAP	P28663	5.32	33,557	80	5	33	−8.897	0.02521
11673	Syntaxin-7	STX7	O70439	5.6	29,821	55	4	22	2.118	0.00333
11737	Dynactin subunit 2	DCTN2	Q99KJ8	5.14	44,117	94	8	21	2.96	0.03845
11800	ADP-ribosyl cyclase 2	BST-1	Q64277	5.49	34,616	85	5	37	−1.516	0.04099
**SEROTONERGIC NEUROTRANSMISSION**										
11967	Tryptophan 5-hydroxylase 1	TPH1	P17532	6.06	51,343	82	7	17	−3.353	0.04087
11670	Serotonin N-acetyltransferase	AA-NAT	O88816	7.01	23,069	56	3	26	1.549	0.00076
**GROWTH FACTORS**										
11646	Secreted protein acidic and rich in cysteine	SPARC	P07214	4.77	34,450	71	5	16	1.388	0.04557
11294	Glypican 6	GPC6	Q9R087	5.32	63,057	86	7	20	2.515	0.01623
11419	Fibroblast growth factor 14	FGF14	P70379	10.11	27,764	74	5	29	−1.61	0.02358
**CELL SURVIVAL PROTEINS**										
11005	TNFAIP3-interacting protein 2	ABIN2	Q99JG7	6.03	49,094	78	8	23	−1.792	0.00449
11105	Cell death regulator Aven	AVEN	Q9D9K3	4.92	37,195	59	5	21	−1.887	0.03471
12856	Regulator of G-protein signaling 10	RGS10	Q9CQE5	6.36	21,151	66	4	44	1.805	8.22E-05
**OTHER**										
12115	Phosphoserine phosphatase	PSPH	Q99LS3	5.81	25,096	56	4	36	1.434	0.01847
11051	Calcium/calmodulin-dependent 3′,5′-cyclic nucleotide phosphodiesterase 1A	PDE1A	Q61481	5.67	64,529	82	7	15	−1.514	0.00902
11988	Galanin-like peptide	GALP	Q810H5	6.41	12,773	61	3	35	−5.496	0.03014
12051	Glyoxalase domain-containing protein 5	GLOD5	Q9D8I3	5.12	16,595	59	4	40	−1.827	0.03871

**Figure 2:**
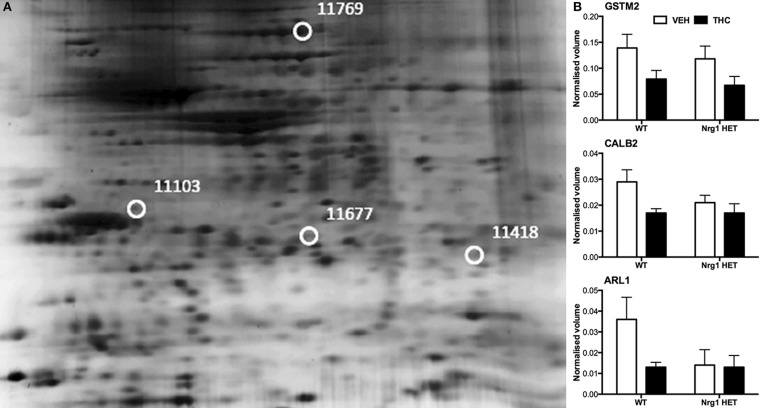
**Adolescent hippocampal proteomics comparing adolescent WT mice treated with vehicle and THC. (A)** Representative 2DE gel images showing positions of significantly altered protein spots, the horizontal axis split by isoelectric point, vertical axis split by molecular weight; [CALB2 (spot 11103); ARL1 (spot 11677); GSTM2 (spot 11418); HSPA4 (spot 11769)]; and **(B)** Exemplar normalized volumes of altered proteins i.e., CALB2, ARL1, and GSTM2.

**Figure 3:**
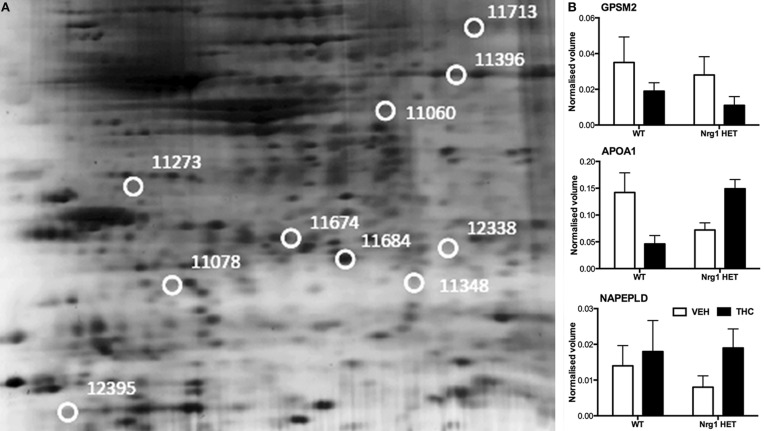
**Adolescent hippocampal proteomics comparing adolescent *Nrg1* HET mice treated with vehicle and THC. (A)** Representative 2D E gel images showing positions of significantly altered protein spots (the horizontal axis split by isoelectric point, vertical axis split by molecular weight; [GPSM2 (spot 11713); APOA1 (spot 11674); NAPEPLD (spot 11060); OTOR (spot 12395)]; and **(B)** exemplar normalized volumes of altered proteins i.e., GPSM2, APOA1 and NAPEPLD.

THC induced changes in 28 spots and 23 spots in WT and *Nrg1* HET mice respectively relative to vehicle-treated mice within the same genotype. From these comparisons, 4 and 10 proteins were identified as being significantly altered by THC exposure in adolescent WT and *Nrg1* HET mice and fold changes are listed in Table 2 (relative to WT mice treated with vehicle) and 3 (relative to *Nrg1* HET mice treated with vehicle) respectively. Figure 2A shows a representative 2D gel image of protein expression in the hippocampus of an adolescent WT mouse administered repeated THC injections. Normalized spot volumes are depicted for 3 representative proteins in Figure 2B, i.e., glutathione S-transferase Mu 2 (GSTM2), calretinin (CALB2) and ADP-ribosylation factor-like protein 1 (ARL1). *Nrg1* HET mice treated with THC displayed a distinct protein expression profile to WT mice exposed to the drug. Figure 3A shows a representative 2D gel image of protein expression in the hippocampus of an adolescent *Nrg1* HET mouse administered repeated THC. Normalized spot volumes of three representative proteins are depicted in Figure 3B, i.e., G-protein-signaling modulator 2 (GPSM2), apolipoprotein A1 (APOA1) and N-acyl-phosphatidylethanolamine-hydrolyzing phospholipase D (NAPEPLD).

**Table 2:** **Adolescent hippocampal proteomics comparing adolescent WT mice treated with vehicle and THC**.

**Spot number**	**Protein name**	**Abbreviation**	**UniProt accession number**	**PI**	**Mass (Da)**	**MWS**	**No. of peptides matched**	**% seq cover**	**Fold change**	***T*-test (p)**
**OXIDATION REGULATION PROTEINS**										
11418	Glutathione S-transferase Mu 2	GSTM2	P15626	6.9	25,717	73	5	20	−1.759	0.02679
11769	Heat shock 70 kDa protein 4	HSPA4	Q61316	5.15	94,133	95	8	14	2.137	0.04305
**OTHER**										
11103	Calretinin	CALB2	Q08331	4.94	31,373	66	4	21	−1.748	0.03045
11677	ADP-ribosylation factor-like protein 1	ARL1	P61211	5.63	20,412	57	3	20	−2.869	0.04051

## Discussion

**Figure 4:**
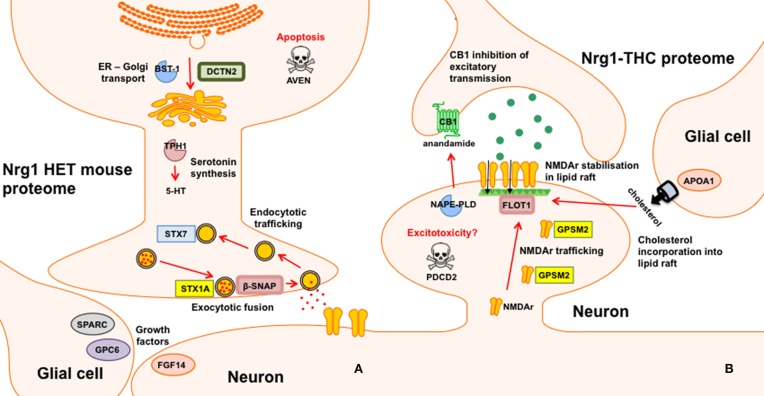
**Schematic representation of altered proteins. (A)** Proteins with different expression in *Nrg1* HET mice relative to WT controls; and **(B)** Proteins with different expression in THC administered *Nrg1* HET mice relative to vehicle treated controls.

*Nrg1* HET mice, displayed altered expression of a number of proteins involved in the vesicular release of neurotransmitters including STX1A, syntaxin 7 (STX7) and β-SNAP; serotonergic neurotransmission including tryptophan 5-hydroxylase 1 (TPH1) and serotonin N-acetyltransferase (AA-NAT); growth factor expression and regulation including secreted protein acidic and rich in cysteine (SPARC) and GPC6, and; cell survival and regulators of inflammatory cytokines including cell death regulator Aven (AVEN), TNFAIP3-interacting protein 2 (ABIN2) and regulator of G-protein signaling 10 (RGS10). We replicated prior findings in rodents without genetic modification showing THC reduced the hippocampal expression of GSTM2 and affected the expression of heat shock proteins (here HSPA4). We also identified novel proteins changed in response to repeated THC exposure, that is, CALB2 and ARL1. Unlike WT mice, *Nrg1* HET mice administered THC displayed altered expression of proteins involved in NMDA receptor trafficking to the synaptic membrane including GPSM2; lipid raft stabilization of receptors at the synaptic membrane including flotillin-1 (FLOT1); homeostatic responses to dampen excessive glutamatergic transmission, including NAPEPLD, and excitotoxicity and apoptosis including programmed cell death protein 2 (PCD2). Figure 4 is a schematic proposing an overview of the proteins found to have altered expression in the current study and their potential functional significance. Proteomics may produce false positives and fold changes < 1.5 should be interpreted cautiously. Nevertheless, these results, while suggestive rather than conclusive, provide a platform for future work to confirm and more fully characterize the role of various novel proteins in the effects of *Nrg1* hypomorphism, THC and *Nrg1*-THC interactions.

### Distinct protein expression in the hippocampus of adolescent *Nrg1* HET and WT mice

Disordered neurotransmission is involved in the pathophysiology of schizophrenia and *NRG1*, a schizophrenia susceptibility gene, regulates neurotransmitter receptor expression and synaptic plasticity (Mei and Xiong, 2008). Here we provide evidence that heterozygous deletion of *Nrg1* alters numerous proteins involved in the transport, fusion and recycling of synaptic vesicles, all processes critical to neurotransmitter release and synaptic function. The soluble N-ethylmaleimide-sensitive fusion attachment protein receptor (SNARE) complex regulates exocytotic release of neurotransmitters from presynaptic terminals and alterations in SNARE mRNA and protein is observed in post-mortem schizophrenia brain (Ramakrishnan et al., 2012). Here we show for the first time that *Nrg1* hypomorphism alters the expression of various SNARE proteins including STX1A, STX7, and β-SNAP.

STX1A was increased almost three-fold in *Nrg1* HET mice relative to WT controls and this change was confirmed by Western blot analysis. Located within the pre-synaptic membrane, STX1A combines with 25 kDa synaptosome-associated protein SNAP25 and vesicle-associated membrane protein 2 to form a complex that drives vesicle and presynaptic membrane fusion necessary for neurotransmitter exocytosis. Concordant with our findings in *Nrg1* HET mice, STX1A is upregulated in the hippocampus and cingulate cortex of schizophrenic patients (Gabriel et al., 1997; Honer et al., 1997; Sokolov et al., 2000; Clark et al., 2007). We also demonstrate here that STX7, a member of an endocytic SNARE complex, was upregulated in *Nrg1* HET mice compared to WT mice. STX7 mediates endocytic trafficking from early endosomes to late endosomes, and is necessary for fusion of late endosomes to lysosomes (Mullock et al., 2000; Nakamura et al., 2000). β-SNAP displayed an almost 9-fold reduction in expression in *Nrg1* HET mice compared to WT mice. β-SNAP belongs to a class of proteins known as SNAPs, which form complexes with SNARE proteins to assist with membrane fusion before being dissociated by the ATPase N-ethylmaleimide-sensitive factor. In contrast to other SNAPs, β-SNAP is localized to neural tissue, including hippocampal cells (Schiavo et al., 1995).

*Nrg1* HET mice also displayed altered expression of several proteins involved in protein transport between the endoplasmic reticulum (ER) and Golgi apparatus. These included β-SNAP, dynactin subunit 2 (DCTN2) and ADP-ribosyl cyclase 2 (BST-1). Transport of protein from the ER to the Golgi is important to protein sorting and the dispatch of protein to cellular locations. An increased expression of DCTN2 was also observed in *Nrg1* HET mice. DCTN2 is a functional subunit of dynactin, a component of the dynein-dynactin system. DCTN2 overexpression inhibits dynactin, and therefore dynein functions such as dynein-dependent maintenance of membrane organelle distribution (Burkhardt et al., 1997). DCTN2 is associated with syntaxin 18, an ER-localized SNARE involved in membrane trafficking between the ER and Golgi (Arasaki et al., 2006). BST-1, which was downregulated in *Nrg1* HET mice, is also implicated in ER to Golgi transport as it suppressed such trafficking in yeast cells (Sompol et al., 2011).

Previous studies suggest that *Nrg1* hypomorphism affects serotonergic neurotransmission, by altering the expression of 5-HT_2A_ receptors and the serotonin transporter in various brain regions of both adolescent and adult mice (Dean et al., 2008; Long et al., 2013). The observation that *Nrg1* HET mice had reduced expression of TPH1 and increased level of AA-NAT is consistent with this notion. TPH1 is one of two isoforms of the enzyme involved in the rate-limiting synthesis of serotonin. Polymorphism in *TPH1* is associated with increased risk for various psychiatric disorders including schizophrenia and bipolar disorder (Saetre et al., 2010; Seifuddin et al., 2012) and varies with neurodevelopment with peaks at PND 21 before decreasing in adulthood (Nakamura et al., 2006). Given the autocrine role of serotonin in guiding the development of serotonergic neurons (Gaspar et al., 2003), reduced TPH1 expression in adolescent *Nrg1* HET mice may reflect aberrant development of serotonergic brain circuitry in these mice. AA-NAT, which converts serotonin to N-acetylserotonin, was also upregulated in the hippocampus of *Nrg1* HET mice. This role of this enzyme is well characterized in the pineal gland due to its involvement in melatonin synthesis and sleep-wake cycles (Zheng and Cole, 2002). However, the function of AA-NAT in other brain regions including the hippocampus is poorly understood. AA-NAT is expressed in a non-diurnal dependent manner in the hippocampus (Uz et al., 2002) and promotes hippocampal neuroprogenitor cell proliferation in mice (Sompol et al., 2011).

Given that *Nrg1* is a neurotrophic factor it is not entirely surprising that *Nrg1* HET mice display altered expression of the growth factor fibroblast growth factor 14 (FGF14) and regulators of growth factor protein expression, including SPARC and GPC6. FGF14 knockout mice, like *Nrg1* HET mice, display locomotor hyperactivity, spatial learning deficits and impaired hippocampal long-term potentiation associated with lowered presynaptic vesicle docking (Wozniak et al., 2007; Xiao et al., 2007). The latter effect is relevant given the altered proteins involved in vesicle docking we observed here in *Nrg1* HET mice. The reduced level of FGF14 might be related to the increased expression of GPC6 and SPARC. Glypicans are heparan sulphate proteoglycans that act as co-receptors for growth factors and modulate fibroblast growth factor signaling (Paine-Saunders et al., 1999; Galli et al., 2003). GPC6 has recently been identified as a gene that confers susceptibility to formal thought disorder in schizophrenia and as a factor released from astrocytes that supports the formation of glutamatergic synapses via GluA1 AMPA receptors (Allen et al., 2012; Wang et al., 2012). Similar to GPC6, SPARC is released from astrocytes and modulates the formation of excitatory synapses and FGF expression (Kucukdereli et al., 2011; Bradshaw, 2012). Taken together these findings suggest a novel link between *Nrg1* and other interrelated growth factor-related proteins FG14, GPC6, and SPARC, worthy of further examination in future studies.

The hippocampus of adolescent *Nrg1* HET mice displayed altered expression of various proteins that influence cell survival and neuroinflammation including AVEN, ABIN2, and RGS10. *Nrg1* exerts neuroprotective effects via inhibiting apoptosis triggered by various challenges (Chen et al., 2011; Li et al., 2012; Woo et al., 2012). AVEN inhibits apoptosis (Chau et al., 2000; Figueroa et al., 2004; Kutuk et al., 2010), therefore the reduction of AVEN in the hippocampus of *Nrg1* HET mice might confer greater vulnerability to apoptosis in these mice. Adolescent *Nrg1* HET mice displayed a trend toward increased expression of the pro-apoptotic and inflammatory cytokine TNF-α in the hippocampus and schizophrenia patients with a missense mutation in the transmembrane domain of *NRG1* show heightened expression of TNF-α from B cells (Marballi et al., 2010; Desbonnet et al., 2012). This is interesting as ABIN-2 and RGS10, two proteins altered in *Nrg1* HET mice, modulate the action of the TNF-α. ABIN-2 prevents TNF-α mediated pro-apoptotic effects and its decreased expression in *Nrg1* HET mice might reflect again a greater propensity to hippocampal apoptosis (Verstrepen et al., 2009). Perhaps as a compensatory mechanism *Nrg1* HET mice showed increased expression of RGS10, a protein which renders cells resistant to TNF-α induced apoptosis (Lee et al., 2012). Future studies are required to confirm whether altered apoptosis and neuroinflammation exists in *Nrg1* HET mice.

### Differential effects of THC on the proteome of *Nrg1* HET mice vs. WT mice

Our prior research shows that *Nrg1* heterozygotes display an altered neurobehavioral response to cannabinoids (Boucher et al., 2007a,b, 2011; Arnold et al., 2012; Long et al., 2013). *Nrg1* mutant mice were more sensitive to the behavioral actions of acute THC compared to WT littermates in a sex-specific manner, with males being selectively affected but not females (Boucher et al., 2007a; Long et al., 2010). In a repeated dosing study, tolerance to cannabinoid-induced hypothermia and locomotor suppression developed more rapidly in *Nrg1* HET than WT mice (Boucher et al., 2011). Conversely, only WT mice developed tolerance to cannabinoid-induced anxiety and *Nrg1* HET mice maintained a persistent anxiogenic response to repeated cannabinoid exposure. Acute and repeated cannabinoid exposure selectively activated expression of Fos transcription factors in the lateral septum of *Nrg1* HET mice but not WT mice (Boucher et al., 2007b, 2011). We also examined whether *Nrg1* hypomorphism confers vulnerability to the neurobehavioral actions of acute or repeated THC exposure in adolescence (Long et al., 2013). THC exposure exacerbated the hyperlocomotor phenotype of *Nrg1* HET mice expressed after withdrawal of the drug. Further, repeated THC administration also promoted differential effects on CB_1_ receptor, 5-HT_2A_ and NMDA receptor binding. Notably adolescent THC exposure selectively increased NMDA receptor expression in the hippocampus of *Nrg1* HET but not WT mice. Given these findings it is perhaps not surprising that the impact of repeated THC treatment as measured by proteomics was quite distinct in *Nrg1* HET mice vs. WT mice, with no overlap in differentially expressed proteins Table 3.

**Table 3:** **Adolescent hippocampal proteomics comparing adolescent *Nrg1* HET mice treated with vehicle and THC**.

**Spot number**	**Protein name**	**Abbreviation**	**UniProt accession number**	**PI**	**Mass (Da)**	**MWS**	**No. of peptides matched**	**% seq cover**	**Fold change**	***T*-test (p)**
**NMDA RECEPTOR PHYSIOLOGY**										
11396	Flotillin-1	FLOT1	O08917	6.71	47,513	82	6	16	1.54	0.001985
11674	Apolipoprotein A-I	APOA1	Q00623	5.64	30,616	73	5	19	2.069	0.003538
11713	G-protein-signaling modulator 2	GPSM2	Q8VDU0	6.49	75,591	77	7	15	−2.648	0.04831
**CELL SURVIVAL/CYTOTOXICITY RELATED PROTEINS**										
12338	Programmed cell death protein 2 (fragment)	PDCD2	Q6RI66	5.24	19,190	66	9	19	1.169	0.0391
11060	N-acyl-phosphatidylethanolamine-hydrolyzing phospholipase D	NAPEPLD	Q8BH82	5.63	45,816	78	6	21	2.334	0.02612
11078	Interleukin-2	IL-2	P04351	4.66	19,400	58	3	34	−1.48	0.03401
**OTHER**										
11273	Translocon-associated protein subunit alpha	SSR1	Q9CY50	4.36	32,065	56	4	20	−3.383	0.02365
11684	Carbonic anhydrase 3	CA3	P16015	6.89	29,366	97		42	−1.293	0.03876
11348	Vacuolar protein-sorting-associated protein 25	VPS25	Q9CQ80	5.97	20,748	62	4	33	−3.211	0.04376
12395	Otoraplin	OTOR	Q9JIE3	4.77	14,328	61	3	21	3.159	0.0296

Nevertheless, our findings show some degree of overlap with previous examinations of THC effects on the rodent brain proteome (Quinn et al., 2008; Colombo et al., 2009; Rubino et al., 2009a; Filipeanu et al., 2011; Wang et al., 2011). THC treatment in adolescent rats modulated proteins regulating oxidative stress such as glutathione S-transferase and heat shock proteins (Quinn et al., 2008). Our results replicate the finding that repeated THC exposure decreased the expression of GSTM2 in the hippocampus (Quinn et al., 2008). GSTM2 catalysis the conjugation of reduced glutathione to electrophilic compounds thereby reducing the deleterious effects of reactive oxygen species (ROS) on cellular lipid, protein and DNA. By reducing levels of GSTM2, THC may render the hippocampus more vulnerable to oxidative stress and this may be linked to the long-term memory impairing effects of cannabinoids (Quinn et al., 2008; Boucher et al., 2009). Phencyclidine, another drug of abuse that promotes schizophrenia-relevant behaviors and cognitive dysfunction, also reduced glutathione levels and antioxidant defense enzymes in the rodent brain (Radonjic et al., 2010; Stojković et al., 2012). Interestingly, copy number variants in genes encoding glutathione S-transferase may be involved in susceptibility to schizophrenia (Rodriguez-Santiago et al., 2010). Here we also showed repeated adolescent THC exposure upregulated the expression of heat shock protein 70 kDa in the hippocampus. Previous studies illustrated effects of rodent THC exposure on heat shock protein 70 kDa, heat shock cognate 71 kDa protein and heat shock 60 kDa protein (Bindukumar et al., 2008; Quinn et al., 2008; Colombo et al., 2009; Rubino et al., 2009a; Filipeanu et al., 2011). Heat shock proteins regulate cellular stress responses and provide protection against oxidative stress (Quinn et al., 2008; Stetler et al., 2010) so their increased expression may signify greater oxidative stress in the hippocampus. Heat shock protein 70 kDa may also serve an autophagic function facilitating the clearance of toxic proteins and assisting in neuronal survival (Stetler et al., 2010).

Adolescent THC exposure decreased hippocampal levels of the calcium-binding protein CALB2. CB_1_ receptors are expressed on calretinin-positive GABA interneurons in the hippocampus (Marsicano and Lutz, 1999; Morozov et al., 2009). THC exposure in C57/BL6 mice increased expression of this protein in the cerebellum (Colombo et al., 2009). Colombo et al. (2009) analyzed CALB2 expression in the membrane whereas we assessed the cytosolic fraction, therefore it remains possible our finding may reflect translocation of the protein from the cytosol to the membrane. We isolated a novel protein, (ARL1), which was downregulated in response to THC exposure. Alcohol and methamphetamine administration similarly alter expression of this protein (Iwazaki et al., 2008; Kobeissy et al., 2008; Kashem et al., 2009). ARL1 is a Ras GTPase involved in retrograde trafficking of endosomes between the Golgi apparatus and the membrane in mammalian cells (Nishimoto-Morita et al., 2009). A THC-induced reduction in ARL1 may then disrupt the distribution of intracellular protein transport in the hippocampus.

Proteins selectively altered in *Nrg1* HET mice treated with THC include those that affect synapse formation and the dynamics of dendritic spines. *Nrg1* is a neurotrophic factor involved in spinogenesis through its modulation of NMDA receptor function (Li et al., 2007; Chen et al., 2008; Barros et al., 2009; Bennett, 2011; Nason et al., 2011). Adolescent THC exposure reduced the density of dendritic spines in the hippocampus via modulation of a number of proteins important to spine dynamics such as PSD-95 and NMDA receptors (Rubino et al., 2009b). *Nrg1* hypomorphism might abnormally increase dendritic spine density in the hippocampus in response to THC as adolescent *Nrg1* HET mice treated with THC displayed increased NMDA receptor binding in the hippocampus (Long et al., 2013). Our proteomic findings indicate altered expression in a number of proteins involved in intracellular trafficking and stabilization of NMDA receptors at the synapse. These include FLOT1, APOA1, and GPSM2.

GPSM2 traffics intracellular NMDA receptors to the synaptic membrane and facilitates spinogenesis by forming a macromolecular complex with NMDA receptors and synapse associated protein 102 (Sans et al., 2005). The reduced level of GPSM2 we observed in THC-treated *Nrg1* HET mice may reflect GPSM2 being incorporated into the macromolecular complex, lowering the observed expression of free, unconjugated GPSM2. Further, *Nrg1* HET mice treated with THC showed a selective increase in FLOT1 expression in the hippocampus, a protein that helps stabilize lipid rafts in the membrane. FLO T1 mediates neurite branching and dendritic spine dynamics in the hippocampus (Swanwick et al., 2010; Raemaekers et al., 2012). It also regulates the formation of glutamatergic synapses and interacts with NMDA receptors, possibly to enhance NMDA receptor clustering or trafficking to the membrane (Allen et al., 2007; Swanwick et al., 2009, 2010). Lipid rafts are constituted by cholesterol and sphingolipids (Mauch et al., 2001; Hering et al., 2003). APOA1, a protein that stimulates cholesterol release from glia, was upregulated in THC exposed *Nrg1* HET mice (Hirsch-Reinshagen et al., 2004; Karten et al., 2005). Therefore, APOA1, by increasing the availability of cholesterol for incorporation into lipid rafts, may have in turn assisted in the molecular events required to stabilize NMDA receptors at the synaptic membrane. Interestingly, APOA1 is altered in schizophrenia brain (Huang et al., 2008).

The increased excitatory transmission mediated by increased NMDA receptors in THC-treated *Nrg1* HET mice might also increase the expression of the apoptotic marker PCD2 and anandamide synthesizing enzyme NAPEPLD (Howlett et al., 2011), proteins reflecting heightened excitotoxicity/apoptosis and a homeostatic attempt to dampen increased NMDA receptor activation respectively. These results are consistent with *Nrg1*-cannabinoid interactions dysregulating the septohippocampal system. Increased excitation in the hippocampus of THC-treated *Nrg1* HET mice might then influence downstream activity of the lateral septum, a region we have repeatedly shown to be selectively activated in *Nrg1* HET mice in response to THC (Boucher et al., 2007b, 2011).

## Conclusions

Using a proteomic approach we have uncovered numerous novel proteins that may be subject to regulation by disturbed *Nrg1* signaling. Our findings also illuminate a potential constellation of molecular changes that may subserve the behavioral abnormalities that are observed in the *Nrg1* transmembrane domain heterozygous mouse as well as their idiosyncratic response to repeated cannabinoid treatment. This may have implications for our overall understanding of genetic vulnerability to schizophrenia and to the exacerbation of psychosis sometimes caused by cannabis.

### Conflict of interest statement

The authors declare that the research was conducted in the absence of any commercial or financial relationships that could be construed as a potential conflict of interest.

## References

[B1] Allen J. A. Halverson-Tamboli R. A. Rasenick M. M. (2007). Lipid raft microdomains and neurotransmitter signalling. Nat. Rev. Neurosci. 8, 128–140 10.1038/nrn2059 17195035

[B2] Allen N. J. Bennett M. L. Foo L. C. Wang G. X. Chakraborty C. Smith S. J. (2012). Astrocyte glypicans 4 and 6 promote formation of excitatory synapses via GluA1 AMPA receptors. Nature 486, 410–414 10.1038/nature11059 22722203 PMC3383085

[B3] Arasaki K. Taniguchi M. Tani K. Tagaya M. (2006). RINT-1 regulates the localization and entry of ZW10 to the syntaxin 18 complex. Mol. Biol. Cell 17, 2780–2788 10.1091/mbc.E05-10-0973 16571679 PMC1474792

[B4] Arnold J. C. Boucher A. A. Karl T. (2012). The yin and yang of cannabis-induced psychosis: the actions of delta 9-tetrahydrocannabinol and cannabidiol in rodent models of Schizophrenia. Curr. Pharm. Des. 18, 5113–5130 10.2174/138161212802884726 22716133

[B5] Ayalew M. Le-Niculescu H. Levey D. F. Jain N. Changala B. Patel S. D. (2012). Convergent functional genomics of schizophrenia: from comprehensive understanding to genetic risk prediction. Mol. Psychiatry 17, 887–905 10.1038/mp.2012.37 22584867 PMC3427857

[B6] Barros C. S. Calabrese B. Chamero P. Roberts A. J. Korzus E. Lloyd K. (2009). Impaired maturation of dendritic spines without disorganization of cortical cell layers in mice lacking *NRG1*/ErbB signaling in the central nervous system. Proc. Natl. Acad. Sci. U.S.A. 106, 4507–4512 10.1073/pnas.0900355106 19240213 PMC2657442

[B7] Bennett M. R. (2011). Schizophrenia: susceptibility genes, dendritic-spine pathology and gray matter loss. Prog. Neurobiol. 95, 275–300 10.1016/j.pneurobio.2011.08.003 21907759

[B8] Bindukumar B. Mahajan S. D. Reynolds J. L. Hu Z. Sykes D. E. Aalinkeel R. (2008). Genomic and proteomic analysis of the effects of cannabinoids on normal human astrocytes. Brain Res. 1191, 1–11 10.1016/j.brainres.2007.10.062 18163980 PMC2821806

[B9] Bjarnadottir M. Misner D. L. Haverfield-Gross S. Bruun S. Helgason V. G. Stefansson H. (2007). Neuregulin1 (*NRG1*) signaling through Fyn modulates NMDA receptor phosphorylation: differential synaptic function in *NRG1* ± knock-outs compared with wild-type mice. J. Neurosci. 27, 4519–4529 10.1523/JNEUROSCI.4314-06.2007 17460065 PMC6672983

[B10] Boucher A. A. Arnold J. C. Duffy L. Schofield P. R. Micheau J. Karl T. (2007a). Heterozygous neuregulin 1 mice are more sensitive to the behavioural effects of Delta9-tetrahydrocannabinol. Psychopharmacology (Berl.) 192, 325–336 10.1007/s00213-007-0721-3 17333138

[B11] Boucher A. A. Hunt G. E. Karl T. Micheau J. McGregor I. S. Arnold J. C. (2007b). Heterozygous neuregulin 1 mice display greater baseline and Delta(9)-tetrahydrocannabinol-induced c-Fos expression. Neuroscience 149, 861–870 10.1016/j.neuroscience.2007.08.020 17905522

[B12] Boucher A. A. Hunt G. E. Micheau J. Huang X. McGregor I. S. Karl T. (2011). The schizophrenia susceptibility gene neuregulin 1 modulates tolerance to the effects of cannabinoids. Int. J. Neuropsychopharmacol. 14, 631–643 10.1017/S146114571000091X 20701826

[B13] Boucher A. A. Vivier L. Metna-Laurent M. Brayda-Bruno L. Mons N. Arnold J. C. (2009). Chronic treatment with Delta(9)-tetrahydrocannabinol impairs spatial memory and reduces zif268 expression in the mouse forebrain. Behav. Pharmacol. 20, 45–55 10.1097/FBP.0b013e3283242f6a 19179850

[B14] Bradshaw A. D. (2012). Diverse biological functions of the SPARC family of proteins. Int. J. Biochem. Cell Biol. 44, 480–488 10.1016/j.biocel.2011.12.021 22249026 PMC3312742

[B15] Burkhardt J. K. Echeverri C. J. Nilsson T. Vallee R. B. (1997). Overexpression of the dynamitin (p50) subunit of the dynactin complex disrupts dynein-dependent maintenance of membrane organelle distribution. J. Cell Biol. 139, 469–484 10.1083/jcb.139.2.469 9334349 PMC2139801

[B16] Casey B. J. Jones R. M. Hare T. A. (2008). The adolescent brain. Ann. N.Y. Acad. Sci. 1124, 111–126 10.1196/annals.1440.010 18400927 PMC2475802

[B17] Chau B. N. Cheng E. H. Kerr D. A. Hardwick J. M. (2000). Aven, a novel inhibitor of caspase activation, binds Bcl-xL and Apaf-1. Mol. Cell 6, 31–40 10.1016/S1097-2765(05)00021-3 10949025

[B18] Chen Y. Zhang M. Li Q. Guo Y. Ding W. Wang L. (2011). Interfering effect and mechanism of neuregulin on experimental dementia model in rats. Behav. Brain Res. 222, 321–325 10.1016/j.bbr.2011.03.063 21473886

[B19] Chen Y. J. Johnson M. A. Lieberman M. D. Goodchild R. E. Schobel S. Lewandowski N. (2008). Type III neuregulin-1 is required for normal sensorimotor gating, memory-related behaviors, and corticostriatal circuit components. J. Neurosci. 28, 6872–6883 10.1523/JNEUROSCI.1815-08.2008 18596162 PMC2728592

[B20] Chesworth R. Yulyaningsih E. Cappas E. Arnold J. Sainsbury A. Karl T. (2012). The response of neuregulin 1 mutant mice to acute restraint stress. Neurosci. Lett. 515, 82–86 10.1016/j.neulet.2012.03.024 22450046

[B21] Chong V. Z. Thompson M. Beltaifa S. Webster M. J. Law A. J. Weickert C. S. (2008). Elevated neuregulin-1 and ErbB4 protein in the prefrontal cortex of schizophrenic patients. Schizophr. Res. 100, 270–280 10.1016/j.schres.2007.12.474 18243664 PMC2746974

[B22] Clark D. Dedova I. Cordwell S. Matsumoto I. (2007). Altered proteins of the anterior cingulate cortex white matter proteome in schizophrenia. Proteomics Clin. Appl. 1, 157–166 10.1002/prca.200600541 21136665

[B23] Colombo G. Rusconi F. Rubino T. Cattaneo A. Martegani E. Parolaro D. (2009). Transcriptomic and proteomic analyses of mouse cerebellum reveals alterations in RasGRF1 expression following *in vivo* chronic treatment with delta 9-tetrahydrocannabinol. J. Mol. Neurosci. 37, 111–122 10.1007/s12031-008-9114-2 18584336

[B24] Dean B. Karl T. Pavey G. Boer S. Duffy L. Scarr E. (2008). Increased levels of serotonin 2A receptors and serotonin transporter in the CNS of neuregulin 1 hypomorphic/mutant mice. Schizophr. Res. 99, 341–349 10.1016/j.schres.2007.10.013 18054201

[B25] Desbonnet L. O'Tuathaigh C. Clarke G. O'Leary C. Petit E. Clarke N. (2012). Phenotypic effects of repeated psychosocial stress during adolescence in mice mutant for the schizophrenia risk gene neuregulin-1: a putative model of gene x environment interaction. Brain Behav. Immun. 26, 660–671 10.1016/j.bbi.2012.02.010 22426432

[B26] Duffy L. Cappas E. Lai D. Boucher A. A. Karl T. (2010). Cognition in transmembrane domain neuregulin 1 mutant mice. Neuroscience 170, 800–807 10.1016/j.neuroscience.2010.07.042 20678553

[B27] Figueroa B. Jr. Chen S. Oyler G. A. Hardwick J. M. Betenbaugh M. J. (2004). Aven and Bcl-xL enhance protection against apoptosis for mammalian cells exposed to various culture conditions. Biotechnol. Bioeng. 85, 589–600 10.1002/bit.10913 14966800

[B28] Filipeanu C. M. Guidry J. J. Leonard S. T. Winsauer P. J. (2011). Delta9-THC increases endogenous AHA1 expression in rat cerebellum and may modulate CB1 receptor function during chronic use. J. Neurochem. 118, 1101–1112 10.1111/j.1471-4159.2011.07391.x 21781118 PMC5443115

[B29] Gabriel S. M. Haroutunian V. Powchik P. Honer W. G. Davidson M. Davies P. (1997). Increased concentrations of presynaptic proteins in the cingulate cortex of subjects with schizophrenia. Arch. Gen. Psychiatry 54, 559–566 10.1001/archpsyc.1997.01830180077010 9193197

[B30] Galli A. Roure A. Zeller R. Dono R. (2003). Glypican 4 modulates FGF signalling and regulates dorsoventral forebrain patterning in Xenopus embryos. Development 130, 4919–4929 10.1242/dev.00706 12930779

[B31] Gaspar P. Cases O. Maroteaux L. (2003). The developmental role of serotonin: news from mouse molecular genetics. Nat. Rev. Neurosci. 4, 1002–1012 10.1038/nrn1256 14618156

[B32] Giedd J. N. Blumenthal J. Jeffries N. O. Castellanos F. X. Liu H. Zijdenbos A. (1999). Brain development during childhood and adolescence: a longitudinal MRI study. Nat. Neurosci. 2, 861–863 10.1038/13158 10491603

[B33] Greenwood T. A. Light G. A. Swerdlow N. R. Radant A. D. Braff D. L. (2012). Association analysis of 94 candidate genes and schizophrenia-related endophenotypes. PLoS ONE 7:e29630 10.1371/journal.pone.0029630 22253750 PMC3258248

[B34] Han S. Yang B. Z. Kranzler H. R. Oslin D. Anton R. Farrer L. A. (2012). Linkage analysis followed by association show *NRG1* associated with cannabis dependence in African Americans. Mol. Psychiatry 72, 637–644 10.1016/j.biopsych.2012.02.038 22520967 PMC3699339

[B35] Hashimoto R. Straub R. E. Weickert C. S. Hyde T. M. Kleinman J. E. Weinberger D. R. (2004). Expression analysis of neuregulin-1 in the dorsolateral prefrontal cortex in schizophrenia. Mol. Psychiatry 9, 299–307 10.1038/sj.mp.4001434 14569272

[B36] Hering H. Lin C. C. Sheng M. (2003). Lipid rafts in the maintenance of synapses, dendritic spines, and surface AMPA receptor stability. J. Neurosci. 23, 3262–3271 12716933 10.1523/JNEUROSCI.23-08-03262.2003 PMC6742299

[B37] Herkenham M. Lynn A. B. Little M. D. Johnson M. R. Melvin L. S. de Costa B. R. (1990). Cannabinoid receptor localization in brain. Proc. Natl. Acad. Sci. U.S.A. 87, 1932–1936 2308954 10.1073/pnas.87.5.1932 PMC53598

[B38] Hirsch-Reinshagen V. Zhou S. Burgess B. L. Bernier L. McIsaac S. A. Chan J. Y. (2004). Deficiency of ABCA1 impairs apolipoprotein E metabolism in brain. J. Biol. Chem. 279, 41197–41207 10.1074/jbc.M407962200 15269218

[B39] Honer W. G. Falkai P. Young C. Wang T. Xie J. Bonner J. (1997). Cingulate cortex synaptic terminal proteins and neural cell adhesion molecule in schizophrenia. Neuroscience 78, 99–110 10.1016/S0306-4522(96)00489-7 9135092

[B40] Hong L. E. Wonodi I. Stine O. C. Mitchell B. D. Thaker G. K. (2008). Evidence of missense mutations on the neuregulin 1 gene affecting function of prepulse inhibition. Biol. Psychiatry 63, 17–23 10.1016/j.biopsych.2007.05.011 17631867 PMC3569848

[B41] Howlett A. C. Reggio P. H. Childers S. R. Hampson R. E. Ulloa N. M. Deutsch D. G. (2011). Endocannabinoid tone versus constitutive activity of cannabinoid receptors. Br. J. Pharmacol. 163, 1329–1343 10.1111/j.1476-5381.2011.01364.x 21545414 PMC3165945

[B42] Huang J. T. Wang L. Prabakaran S. Wengenroth M. Lockstone H. E. Koethe D. (2008). Independent protein-profiling studies show a decrease in apolipoprotein A1 levels in schizophrenia CSF, brain and peripheral tissues. Mol. Psychiatry 13, 1118–1128 10.1038/sj.mp.4002108 17938634

[B43] Iwazaki T. McGregor I. S. Matsumoto I. (2008). Protein expression profile in the amygdala of rats with methamphetamine-induced behavioral sensitization. Neurosci. Lett. 435, 113–119 10.1016/j.neulet.2008.02.025 18346852

[B44] Karl T. Duffy L. Scimone A. Harvey R. P. Schofield P. R. (2007). Altered motor activity, exploration and anxiety in heterozygous neuregulin 1 mutant mice: implications for understanding schizophrenia. Genes Brain Behav. 6, 677–687 10.1111/j.1601-183X.2006.00298.x 17309661

[B45] Karten B. Hayashi H. Francis G. A. Campenot R. B. Vance D. E. Vance J. E. (2005). Generation and function of astroglial lipoproteins from Niemann-Pick type C1-deficient mice. Biochem. J. 387, 779–788 10.1042/BJ20041694 15544574 PMC1135009

[B46] Kashem M. A. Sarker R. Des Etages H. Machaalani R. King N. McGregor I. S. (2009). Comparative proteomics in the corpus callosal sub-regions of postmortem human brain. Neurochem. Int. 55, 483–490 10.1016/j.neuint.2009.04.017 19433127

[B47] Kobeissy F. H. Warren M. W. Ottens A. K. Sadasivan S. Zhang Z. Gold M. S. (2008). Psychoproteomic analysis of rat cortex following acute methamphetamine exposure. J. Proteome Res. 7, 1971–1983 10.1021/pr800029h 18452277

[B48] Kucukdereli H. Allen N. J. Lee A. T. Feng A. Ozlu M. I. Conatser L. M. (2011). Control of excitatory CNS synaptogenesis by astrocyte-secreted proteins Hevin and SPARC. Proc. Natl. Acad. Sci. U.S.A. 108, E440–E449 10.1073/pnas.1104977108 21788491 PMC3156217

[B49] Kutuk O. Temel S. G. Tolunay S. Basaga H. (2010). Aven blocks DNA damage-induced apoptosis by stabilising Bcl-xL. Eur. J. Cancer 46, 2494–2505 10.1016/j.ejca.2010.06.011 20619636

[B50] Lee J. K. Chung J. Druey K. M. Tansey M. G. (2012). RGS10 exerts a neuroprotective role through the PKA/c-AMP response-element (CREB) pathway in dopaminergic neuron-like cells. J. Neurochem. 122, 333–343 10.1111/j.1471-4159.2012.07780.x 22564151 PMC3435458

[B51] Li B. Woo R. S. Mei L. Malinow R. (2007). The neuregulin-1 receptor erbB4 controls glutamatergic synapse maturation and plasticity. Neuron 54, 583–597 10.1016/j.neuron.2007.03.028 17521571 PMC2031848

[B52] Li Y. Lein P. J. Liu C. Bruun D. A. Giulivi C. Ford G. D. (2012). Neuregulin-1 is neuroprotective in a rat model of organophosphate-induced delayed neuronal injury. Toxicol. Appl. Pharmacol. 262, 194–204 10.1016/j.taap.2012.05.001 22583949 PMC3607352

[B53] Long L. E. Chesworth R. Arnold J. C. Karl T. (2010). A follow-up study: acute behavioural effects of Delta(9)-THC in female heterozygous neuregulin 1 transmembrane domain mutant mice. Psychopharmacology (Berl.) 211, 277–289 10.1007/s00213-010-1896-6 20571781

[B54] Long L. E. Chesworth R. Huang X. F. McGregor I. S. Arnold J. C. Karl T. (2013). Transmembrane domain *Nrg1* mutant mice show altered susceptibility to the neurobehavioural actions of repeated THC exposure in adolescence. Int. J. Neuropsychopharmacol. 16, 163–175 10.1017/S1461145711001854 22226049

[B55] Marballi K. Cruz D. Thompson P. Walss-Bass C. (2012). Differential neuregulin 1 cleavage in the prefrontal cortex and hippocampus in schizophrenia and bipolar disorder: preliminary findings. PLoS ONE 7:e36431 10.1371/journal.pone.0036431 22590542 PMC3349664

[B56] Marballi K. Quinones M. P. Jimenez F. Escamilla M. A. Raventos H. Soto-Bernardini M. C. (2010). *In vivo* and *in vitro* genetic evidence of involvement of neuregulin 1 in immune system dysregulation. J. Mol. Med. (Berl.) 88, 1133–1141 10.1007/s00109-010-0653-y 20625696 PMC2976656

[B57] Marsicano G. Lutz B. (1999). Expression of the cannabinoid receptor CB1 in distinct neuronal subpopulations in the adult mouse forebrain. Eur. J. Neurosci. 11, 4213–4225 10.1046/j.1460-9568.1999.00847.x 10594647

[B58] Mauch D. H. Nagler K. Schumacher S. Goritz C. Muller E. C. Otto A. (2001). CNS synaptogenesis promoted by glia-derived cholesterol. Science 294, 1354–1357 10.1126/science.294.5545.1354 11701931

[B59] Mei L. Xiong W. C. (2008). Neuregulin 1 in neural development, synaptic plasticity and schizophrenia. Nat. Rev. Neurosci. 9, 437–452 10.1038/nrn2392 18478032 PMC2682371

[B60] Morozov Y. M. Torii M. Rakic P. (2009). Origin, early commitment, migratory routes, and destination of cannabinoid type 1 receptor-containing interneurons. Cereb. Cortex 19(Suppl. 1), i78–i89 10.1093/cercor/bhp028 19346272 PMC3584650

[B61] Mullock B. M. Smith C. W. Ihrke G. Bright N. A. Lindsay M. Parkinson E. J. (2000). Syntaxin 7 is localized to late endosome compartments, associates with Vamp 8, and Is required for late endosome-lysosome fusion. Mol. Biol. Cell 11, 3137–3153 10982406 10.1091/mbc.11.9.3137 PMC14981

[B62] Nakamura K. Sugawara Y. Sawabe K. Ohashi A. Tsurui H. Xiu Y. (2006). Late developmental stage-specific role of tryptophan hydroxylase 1 in brain serotonin levels. J. Neurosci. 26, 530–534 10.1523/JNEUROSCI.1835-05.2006 16407550 PMC6674418

[B63] Nakamura N. Yamamoto A. Wada Y. Futai M. (2000). Syntaxin 7 mediates endocytic trafficking to late endosomes. J. Biol. Chem. 275, 6523–6529 10.1074/jbc.275.9.6523 10692457

[B64] Nason M. W. Jr. Adhikari A. Bozinoski M. Gordon J. A. Role L. W. (2011). Disrupted activity in the hippocampal-accumbens circuit of type III neuregulin 1 mutant mice. Neuropsychopharmacology 36, 488–496 10.1038/npp.2010.180 20927045 PMC3005939

[B65] Nishimoto-Morita K. Shin H. W. Mitsuhashi H. Kitamura M. Zhang Q. Johannes L. (2009). Differential effects of depletion of ARL1 and ARFRP1 on membrane trafficking between the trans-Golgi network and endosomes. J. Biol. Chem. 284, 10583–10592 10.1074/jbc.M900847200 19224922 PMC2667745

[B66] O'Tuathaigh C. M. Babovic D. O'Sullivan G. J. Clifford J. J. Tighe O. Croke D. T. (2007). Phenotypic characterization of spatial cognition and social behavior in mice with ‘knockout’ of the schizophrenia risk gene neuregulin 1. Neuroscience 147, 18–27 10.1016/j.neuroscience.2007.03.051 17512671

[B67] O'Tuathaigh C. M. O'Connor A. M. O'Sullivan G. J. Lai D. Harvey R. Croke D. T. (2008). Disruption to social dyadic interactions but not emotional/anxiety-related behaviour in mice with heterozygous ‘knockout’ of the schizophrenia risk gene neuregulin-1. Prog. Neuropsychopharmacol. Biol. Psychiatry 32, 462–466 10.1016/j.pnpbp.2007.09.018 17980471

[B68] Paine-Saunders S. Viviano B. L. Saunders S. (1999). GPC6, a novel member of the glypican gene family, encodes a product structurally related to GPC4 and is colocalized with GPC5 on human chromosome 13. Genomics 57, 455–458 10.1006/geno.1999.5793 10329016

[B69] Quinn H. R. Matsumoto I. Callaghan P. D. Long L. E. Arnold J. C. Gunasekaran N. (2008). Adolescent rats find repeated Delta(9)-THC less aversive than adult rats but display greater residual cognitive deficits and changes in hippocampal protein expression following exposure. Neuropsychopharmacology 33, 1113–1126 10.1038/sj.npp.1301475 17581536

[B70] Radonjic N. V. Knezevic I. D. Vilimanovich U. Kravic-Stevovic T. Marina L. V. Nikolic T. (2010). Decreased glutathione levels and altered antioxidant defense in an animal model of schizophrenia: long-term effects of perinatal phencyclidine administration. Neuropharmacology 58, 739–745 10.1016/j.neuropharm.2009.12.009 20036264

[B71] Raemaekers T. Peric A. Baatsen P. Sannerud R. Declerck I. Baert V. (2012). ARF6-mediated endosomal transport of Telencephalin affects dendritic filopodia-to-spine maturation. EMBO J. 31, 3252–3269 10.1038/emboj.2012.182 22781129 PMC3411082

[B72] Ramakrishnan N. A. Drescher M. J. Drescher D. G. (2012). The SNARE complex in neuronal and sensory cells. Mol. Cell. Neurosci. 50, 58–69 10.1016/j.mcn.2012.03.009 22498053 PMC3570063

[B73] Rodriguez-Santiago B. Brunet A. Sobrino B. Serra-Juhe C. Flores R. Armengol L. (2010). Association of common copy number variants at the glutathione S-transferase genes and rare novel genomic changes with schizophrenia. Mol. Psychiatry 15, 1023–1033 10.1038/mp.2009.53 19528963

[B74] Roussos P. Giakoumaki S. G. Adamaki E. Bitsios P. (2011). The influence of schizophrenia-related neuregulin-1 polymorphisms on sensorimotor gating in healthy males. Biol. Psychiatry 69, 479–486 10.1016/j.biopsych.2010.09.009 21035784

[B75] Rubino T. Realini N. Braida D. Alberio T. Capurro V. Vigano D. (2009a). The depressive phenotype induced in adult female rats by adolescent exposure to THC is associated with cognitive impairment and altered neuroplasticity in the prefrontal cortex. Neurotox. Res. 15, 291–302 10.1007/s12640-009-9031-3 19384563

[B76] Rubino T. Realini N. Braida D. Guidi S. Capurro V. Vigano D. (2009b). Changes in hippocampal morphology and neuroplasticity induced by adolescent THC treatment are associated with cognitive impairment in adulthood. Hippocampus 19, 763–772 10.1002/hipo.20554 19156848

[B77] Saetre P. Lundmark P. Wang A. Hansen T. Rasmussen H. B. Djurovic S. (2010). The tryptophan hydroxylase 1 (TPH1) gene, schizophrenia susceptibility, and suicidal behavior: a multi-centre case-control study and meta-analysis. Am. J. Med. Genet. B Neuropsychiatr. Genet. 153B, 387–396 10.1002/ajmg.b.30991 19526457

[B78] Sans N. Wang P. Y. Du Q. Petralia R. S. Wang Y. X. Nakka S. (2005). mPins modulates PSD-95 and SAP102 trafficking and influences NMDA receptor surface expression. Nat. Cell Biol. 7, 1179–1190 10.1038/ncb1325 16299499

[B79] Schiavo G. Gmachl M. J. Stenbeck G. Sollner T. H. Rothman J. E. (1995). A possible docking and fusion particle for synaptic transmission. Nature 378, 733–736 10.1038/378733a0 7501022

[B80] Seifuddin F. Mahon P. B. Judy J. Pirooznia M. Jancic D. Taylor J. (2012). Meta-analysis of genetic association studies on bipolar disorder. Am. J. Med. Genet. B Neuropsychiatr. Genet. 159B, 508–518 10.1002/ajmg.b.32057 22573399 PMC3582382

[B81] Sokolov B. P. Tcherepanov A. A. Haroutunian V. Davis K. L. (2000). Levels of mRNAs encoding synaptic vesicle and synaptic plasma membrane proteins in the temporal cortex of elderly schizophrenic patients. Biol. Psychiatry 48, 184–196 10924661 10.1016/s0006-3223(00)00875-1

[B82] Sompol P. Liu X. Baba K. Paul K. N. Tosini G. Iuvone P. M. (2011). N-acetylserotonin promotes hippocampal neuroprogenitor cell proliferation in sleep-deprived mice. Proc. Natl. Acad. Sci. U.S.A. 108, 8844–8849 10.1073/pnas.1105114108 21555574 PMC3102377

[B83] Spear L. P. (2000). The adolescent brain and age-related behavioral manifestations. Neurosci. Biobehav. Rev. 24, 417–463 10.1016/S0149-7634(00)00014-2 10817843

[B84] Spencer J. R. Darbyshire K. M. Boucher A. A. Arnold J. C. (2012). Adolescent neuregulin 1 heterozygous mice display enhanced behavioural sensitivity to methamphetamine. Prog. Neuropsychopharmacol. Biol. Psychiatry 39, 376–381 10.1016/j.pnpbp.2012.07.014 22850204

[B85] Stefansson H. Sigurdsson E. Steinthorsdottir V. Bjornsdottir S. Sigmundsson T. Ghosh S. (2002). Neuregulin 1 and susceptibility to schizophrenia. Am. J. Hum. Genet. 71, 877–892 10.1086/342734 12145742 PMC378543

[B86] Stetler R. A. Gan Y. Zhang W. Liou A. K. Gao Y. Cao G. (2010). Heat shock proteins: cellular and molecular mechanisms in the central nervous system. Prog. Neurobiol. 92, 184–211 10.1016/j.pneurobio.2010.05.002 20685377 PMC2939168

[B87] Stojković T. Radonjić N. V. Velimirović M. Jevtić G. Popović V. Doknić M. (2012). Risperidone reverses phencyclidine induced decrease in glutathione levels and alterations of antioxidant defense in rat brain. Prog. Neuropsychopharmacol. Biol. Psychiatry 39, 192–199 10.1016/j.pnpbp.2012.06.013 22735395

[B88] Swanwick C. C. Shapiro M. E. Vicini S. Wenthold R. J. (2010). Flotillin-1 mediates neurite branching induced by synaptic adhesion-like molecule 4 in hippocampal neurons. Mol. Cell. Neurosci. 45, 213–225 10.1016/j.mcn.2010.06.012 20600927

[B89] Swanwick C. C. Shapiro M. E. Yi Z. Chang K. Wenthold R. J. (2009). NMDA receptors interact with flotillin-1 and -2, lipid raft-associated proteins. FEBS Lett. 583, 1226–1230 10.1016/j.febslet.2009.03.017 19298817

[B90] Tsou K. Brown S. Sanudo-Pena M. C. Mackie K. Walker J. M. (1998). Immunohistochemical distribution of cannabinoid CB1 receptors in the rat central nervous system. Neuroscience 83, 393–411 10.1016/S0306-4522(97)00436-3 9460749

[B91] Uz T. Qu T. Sugaya K. Manev H. (2002). Neuronal expression of arylalkylamine N-acetyltransferase (AANAT) mRNA in the rat brain. Neurosci. Res. 42, 309–316 10.1016/S0168-0102(02)00011-1 11985883

[B92] Verstrepen L. Carpentier I. Verhelst K. Beyaert R. (2009). ABINs: A20 binding inhibitors of NF-kappa B and apoptosis signaling. Biochem. Pharmacol. 78, 105–114 10.1016/j.bcp.2009.02.009 19464428

[B93] Vullhorst D. Neddens J. Karavanova I. Tricoire L. Petralia R. S. McBain C. J. (2009). Selective expression of ErbB4 in interneurons, but not pyramidal cells, of the rodent hippocampus. J. Neurosci. 29, 12255–12264 10.1523/JNEUROSCI.2454-09.2009 19793984 PMC2774835

[B94] Wang J. Yuan W. Li M. D. (2011). Genes and pathways co-associated with the exposure to multiple drugs of abuse, including alcohol, amphetamine/methamphetamine, cocaine, marijuana, morphine, and/or nicotine: a review of proteomics analyses. Mol. Neurobiol. 44, 269–286 10.1007/s12035-011-8202-4 21922273

[B95] Wang K. S. Zhang Q. Liu X. Wu L. Zeng M. (2012). PKNOX2 is associated with formal thought disorder in schizophrenia: a meta-analysis of two genome-wide association studies. J. Mol. Neurosci. 48, 265–272 10.1007/s12031-012-9787-4 22648509

[B96] Weickert C. S. Tiwari Y. Schofield P. R. Mowry B. J. Fullerton J. M. (2012). Schizophrenia-associated HapICE haplotype is associated with increased *NRG1* type III expression and high nucleotide diversity. Transl. Psychiatry 2:e104 10.1038/tp.2012.25 22832904 PMC3337073

[B97] Woo R. S. Lee J. H. Kim H. S. Baek C. H. Song D. Y. Suh Y. H. (2012). Neuregulin-1 protects against neurotoxicities induced by Swedish amyloid precursor protein via the ErbB4 receptor. Neuroscience 202, 413–423 10.1016/j.neuroscience.2011.11.026 22186019

[B98] Wozniak D. F. Xiao M. Xu L. Yamada K. A. Ornitz D. M. (2007). Impaired spatial learning and defective theta burst induced LTP in mice lacking fibroblast growth factor 14. Neurobiol. Dis. 26, 14–26 10.1016/j.nbd.2006.11.014 17236779 PMC2267915

[B99] Xiao M. Xu L. Laezza F. Yamada K. Feng S. Ornitz D. M. (2007). Impaired hippocampal synaptic transmission and plasticity in mice lacking fibroblast growth factor 14. Mol. Cell. Neurosci. 34, 366–377 10.1016/j.mcn.2006.11.020 17208450

[B100] Zheng W. Cole P. A. (2002). Serotonin N-acetyltransferase: mechanism and inhibition. Curr. Med. Chem. 9, 1187–1199 10.2174/0929867023370013 12052171

